# Size Selectivity of Square-Mesh Codends in Stownet for *Coilia mystus* in the South China Sea

**DOI:** 10.3390/ani15243571

**Published:** 2025-12-11

**Authors:** Lei Yan, Qingcheng Zhu, Peng Zhang, Jie Li, Bingzhong Yang, Teng Wang, Chuanxiang Hua

**Affiliations:** 1 College of Marine Living Resource Sciences and Management, Shanghai Ocean University, Shanghai 201306, China; yanlei@scfri.ac.cn (L.Y.);; 2 Key Laboratory for Sustainable Utilization of Open-Sea Fishery, Ministry of Agriculture and Rural Affairs, South China Sea Fisheries Research Institute, Chinese Academy of Fishery Sciences, Guangzhou 510300, China

**Keywords:** stownet, square-mesh, codend, size selectivity, *Coilia mystus*

## Abstract

Stownet is one of the most important items of fishing gear for catching *Coilia mystus* in China’s coastal waters. To improve the selectivity of stownet for *C. mystus*, a series of selectivity experiments were conducted using the covered net method with 4 square-mesh codends of different mesh sizes of 25 mm, 30 mm, 35 mm, and 40 mm. The selectivity curves of *C. mystus* under different mesh sizes were fitted using the Logistic and Richards models, and the selectivity parameters were compared among codends. The results showed that the 50% selection length (L50) increased with mesh size, reaching 105.83 mm, 109.02 mm, 114.36 mm, and 116.66 mm, respectively. The proportion of juvenile *C. mystus* in the codends decreased correspondingly, indicating that larger mesh sizes improved the selectivity of the square-mesh codend. Based on the linear relationship between L50 and mesh size, the minimum mesh size for *C. mystus* was estimated to be 43.81 mm. Considering the management regulation of the minimum landing size (120 mm) for *C. mystus*, the L50 obtained for the 40 mm square-mesh was still below this standard, suggesting that the selectivity of the current codend requires further optimization to promote the sustainable utilization of *C. mystus* resources.

## Simple Summary

To protect an important fish species in the South China Sea, *Coilia mystus*, this study aimed to identify a suitable mesh size that allows juvenile fish to escape and reduces bycatch. A series of selectivity experiments were conducted with mesh sizes of 25, 30, 35, and 40 mm. The results showed that larger mesh sizes captured fewer juvenile fish, indicating improved selectivity. Based on our analysis, the minimum mesh size needed to meet management regulations is approximately 44 mm. Overall, this study confirms that increasing mesh size can enhance codend selectivity, but the currently used 40 mm mesh still requires further improvement to better protect *C. mystus* and support sustainable fisheries.

## 1. Introduction

*Coilia mystus*, belonging to the order *Clupeiformes*, family *Engraulidae*, and genus *Coilia*, commonly known as tapertail anchovy or “Fengwei fish” in Chinese, is an important commercial species in the Pearl River Estuary of the South China Sea [1]. Recent regional fishery statistics indicate that the annual catch of *C. mystus* in the South China Sea is approximately 34,000 tonnes, and the commercial price typically ranges from ¥30 to ¥60 per kilogram, underscoring its high economic value and importance to coastal stownet fisheries [2]. It has been included in the National Key Protected Aquatic Germplasm Resources List. As a key species in the estuarine food web, *C. mystus* plays an essential intermediary role in energy transfer, and its stock dynamics have been incorporated into the ecological restoration monitoring system of the Pearl River Estuary [3]. This species is the dominant catch in the two-stick stownet fishery in the South China Sea, accounting for more than 50% of the total catch [4,5,6]. According to our latest field survey data (2023–2024), there are approximately 900 operating vessels (fishing units) engaged in the two-stick stownet fishery in the South China Sea, which are mainly distributed in the coastal areas of Zhanjiang (Guangdong Province) and Haikou (Hainan Province). Most of these vessels are small fishing boats with a total length of less than 12 m, and the annual operating period is from March to November. However, the small mesh size commonly used in these nets results in a high proportion of juvenile fish in the catch, which can severely impact juvenile fish resources. To promote the sustainable development of coastal fisheries, the former Ministry of Agriculture issued in 2013 a notice on the minimum mesh sizes for approved and transitional fishing gears. The notice stipulated a minimum mesh size of 35 mm for stownet in the South China Sea, but it did not include specific management regulations for square mesh.

Square-mesh codends have demonstrated clear advantages in facilitating the escape of juvenile fish and effectively reducing the bycatch of non-target species [7,8,9,10]. In contrast, diamond meshes—although characterized by high flexibility, good extensibility, and structural stability [11,12,13,14]—tend to close under tension as catches accumulate during towing, thereby diminishing their selectivity. By comparison, square meshes can maintain a more open configuration throughout the fishing process, exhibiting superior selective performance.

In recent years, considerable attention has been devoted to understanding the role of square meshes in improving gear selectivity. Herrmann et al. [15] reported that the position of square-mesh escape panels in trawl nets critically affects the release efficiency of Atlantic cod. Similarly, Cuende et al. [16] demonstrated that incorporating square meshes in Basque otter trawls significantly enhances size selectivity and reduces the capture of non-target individuals. Studies by Huang et al. and Zhang et al. further confirmed that square-mesh structures promote the escape of juvenile or fusiform and round-bodied fish species [17,18].

Although the selectivity of square-mesh codends has been investigated in various regions, differences in target species and local ecological conditions have led to variations in recommended minimum mesh sizes. For instance, Huang et al. (2023) proposed a 40 mm square mesh for Larimichthys polyactis in the Haizhou Bay of the Yellow Sea [13]; while Song et al. (2017), who focused on bottom-trawl fisheries in the East China Sea, recommended that the square-mesh codend size should be no less than 55 mm for the same species [19]. However, research on the application of square meshes in stownet in the South China Sea remains scarce. To address this gap, the present study focuses on the two-stick stownet fishery in the South China Sea, conducting selectivity experiments on square-mesh codends with different mesh sizes to evaluate their efficiency in releasing *C. mystus*. The findings aim to identify an appropriate minimum mesh size and provide a scientific basis for the sustainable management of stownet fisheries in the region.

## 2. Materials and Methods

### 2.1. Fishing Experiments

The selectivity experiments were conducted from 5 to 24 September 2019 in the northern South China Sea, near the “Huangmaohai” Estuary. The fishing operations were carried out at positions between 22°08′ N and 22°09′ N, and 113°05′ E and 113°06′ E, over muddy-sandy bottoms at depths ranging from 7 to 8 m, using a fixed gear operation.

The experimental vessel used in this study was the Yue Xinhui 41008, registered at Yamen Port. The vessel was wooden-hulled, with an overall length of 12.00 m, a beam of 2.85 m, and a depth of 0.83 m. It had a gross tonnage of 8.0 GT and a net tonnage of 3.0 t. The vessel was powered by two main engines with a combined output of 19.4 kW (9.7 kW each) and was equipped with standard navigation and safety instruments, including a collision-avoidance radar and a GPS navigation system.

The fishing gear used in this study was a two-stick stownet. The main dimensions of the net were 160.00 m × 67.89 m, corresponding to a stretched mesh perimeter of 160.00 m at the mouth and a total stretched length of 67.89 m along the longitudinal axis. The netting was composed of two main parts: the body netting and the codend. The body section consisted of 24 panels, all made of knotted netting. The body netting and the codend were woven separately; after the body netting was completed, it was stretched and shaped before being joined to the codend. A detailed layout of the netting design is provided in reference [5].

**Figure 1:**
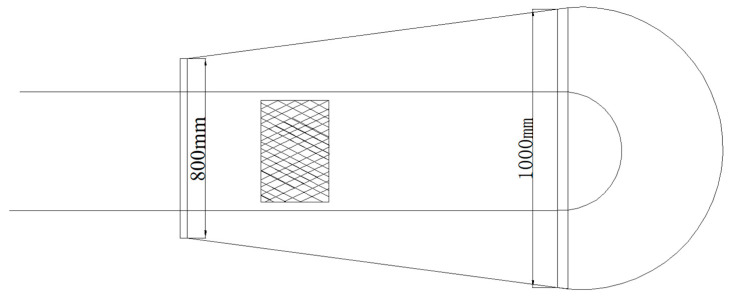
Schematic Diagram of the codend and cover net.

The cover net method was employed to investigate the selectivity of the codends. In this method, a small-mesh cover net was installed outside the experimental codend to collect escaping fish. The mesh size of the cover net was 15 mm, with a stretched perimeter 1.2 times and a stretched length 1.5 times that of the codend. To minimize the covering effect inherent in this method, two iron rings with diameters of 800 mm and 1000 mm were mounted inside the cover net, with a spacing of 2.7 m between them (Figure 1).

**Figure 2:**
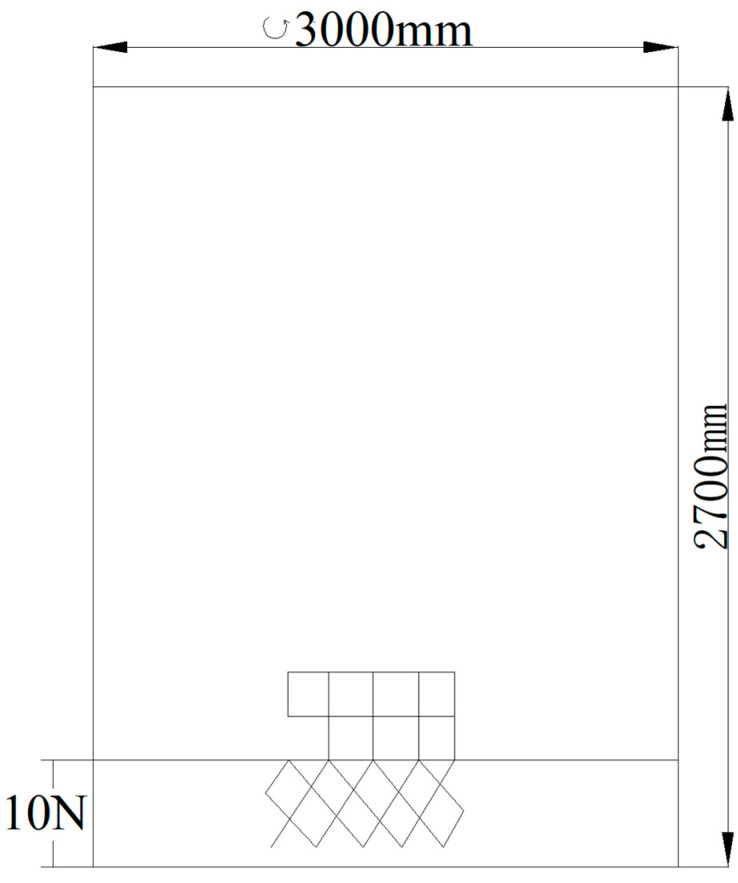
Schematic diagram of the square mesh codend.

4 experimental codends made of square meshes with different mesh sizes (25 mm, 30 mm, 35 mm, and 40 mm) were used. The netting material was polyethylene (PE 36 tex × 2 × 3). To facilitate attachment to the body netting, a short section of diamond-mesh netting (10 meshes in length) with the same mesh size was added to the end of each square-mesh codend, as illustrated in Figure 2. The 4 codends were designated F25, F30, F35, and F40 according to their respective mesh sizes (Table 1).

### 2.2. Data Analysis

#### 2.2.1. Data Collection

During the experiment, only 1 type of experimental codend was used in each fishing operation, and 6 valid operations were conducted for each codend type. The nets were set and retrieved according to tidal conditions, with each operation lasting approximately 4 h. After retrieval, the catches from the codend and the cover net were collected separately for analysis. Sampling procedures followed the Specifications for Oceanographic Survey—Part 6: Marine Biological Survey (GB/T 12763.6–2007) [20].

On the day of sampling, all catches were sorted and identified to species. For each species, body length and body weight were measured. 50 individuals were randomly selected from each species for measurement; when fewer than 50 individuals were available, all were measured. At the sampling stage, no target or key species was pre-assigned, and all species were processed using the same sampling protocol. *C. mystus* was later identified as the key species for selectivity analysis based on its dominant presence in the catch and its high IRI value during post-processing of the data. The total number and total weight of each species were also recorded. All experimental fishes used in this study were collected from routine commercial fishing operations and were already dead upon landing. Therefore, no experimental procedures caused additional mortality or serious illness to the fish. No adverse events occurred during the study.

#### 2.2.2. Dominant Species Determination

The dominant species in the catches from codends and cover nets with different mesh sizes were identified using the index of relative importance (IRI) proposed by Cheng [21]. The IRI was calculated as:

(1) IRI=(N+W)F

where *N* is the percentage of individuals by number, *W* is the percentage by weight, and *F* is the frequency of occurrence (percentage of fishing operations in which the species appeared). Species with IRI values greater than 1000 were considered dominant in the catch composition.

#### 2.2.3. Selectivity Models and Parameters Estimation

The Logistic curve is symmetric around the 50% retention length (L50). However, empirical studies have shown that the selectivity curve is asymmetric in many cases, making it necessary to adopt the asymmetric Richards curve for comparative analysis. The selectivity of the tested codends for the target species was analyzed using both the Logistic and Richards models for comparison [22,23].

The Logistic selectivity curve is expressed as:

(2) $$r(l)=\frac{\exp(a+bl)}{1+\exp(a+bl)}$$

The selectivity parameters are calculated as follows:

(3) $$L50=-\frac{a}{b}$$

(4) $$SR=\frac{2\ln3}{b}$$

The Richards selectivity curve is expressed as:

(5) $$r(l)={\left[\frac{\exp(a+bl)}{1+\exp(a+bl)}\right]}^{1/\delta }$$

The selectivity parameters for the Richards model are calculated as:

(6) $$L50=\frac{-\ln(2^{\delta }-1)-a}{b}$$

(7) $$SR=\frac{\delta \ln3+\ln(1-0.25^{\delta })-\ln(1-0.75^{\delta })}{b}$$

where *r*(*l*) is the selection probability for fish of length *l*, and *a* and *b* are the model parameters, and L50 is the fish length at 50% retention probability, SR is the selection range (difference between the lengths retained at 25% and 75%), and *δ* is the shape parameter of the Richards curve.

All parameters, including *a*, *b*, and *δ* in both the Logistic curve and the Richards curve, can be estimated by maximizing the following log-likelihood function [24,25]:

(8) $$l(\theta )=\ln(L)=\sum_{i}\left\{Nni\cdot \ln[r(l_{i})]+Nci\cdot \ln[1-r(l_{i})]\right\}$$

where *Nni* is the catch number of *C. mystus* in the tested codends and *Nni* is the catch number in the relative covers. To maximize Equation (8), we used SOLVER in Microsoft Excel-2019 (Version 16.0.17830.20166, 64-bit). The standard errors of all derived parameters were obtained via the parametric bootstrap method (a commonly used approach in fishery selectivity analysis for error estimation).

In both the Logistic curve and the Richards curve, the standard errors of parameters including *a*, *b*, *δ*, L50 and SR could be obtained by the estimated method.

#### 2.2.4. Relationship Between L50 and Mesh Size

Assuming that fish maintain a similar body shape during growth, and under identical fishing conditions, the relationship between L50 and mesh size can be described based on the principle of geometric similarity. According to this principle, L50 increases proportionally with increasing mesh size. Both domestic and international studies have shown that this relationship is generally linear [26], and can be expressed as:

(9) L50=a+b×m

where *a* and *b* are constants, and m is the mesh size (mm).

## 3. Results

### 3.1. Catch Composition

During the experimental period, a total of 24 valid fishing operations were conducted, with 6 operations performed for each of the 4 square-mesh codends (F25, F30, F35, and F40). In total, 5724 individuals were measured, with a combined catch weight of 57.61 kg.

Species identification revealed 22 species in total, including 21 fish species belonging to 1 class, 6 orders, 11 families, and 19 genera, and one shrimp species belonging to 1 class, 1 order, and 1 family.

The dominant species identified based on the index of relative importance (IRI) are listed in Table 2. The dominant species varied slightly among codends with different mesh sizes; however, *C. mystus* was the overwhelmingly dominant species, accounting for 76.33–85.96% of the total number and 55.91–72.62% of the total catch weight. Based on the IRI values, *C. mystus* was selected as the indicator species for comparing the selective performance among different codends.

### 3.2. Length Distribution of *C*. *mystus*

**Figure 3:**
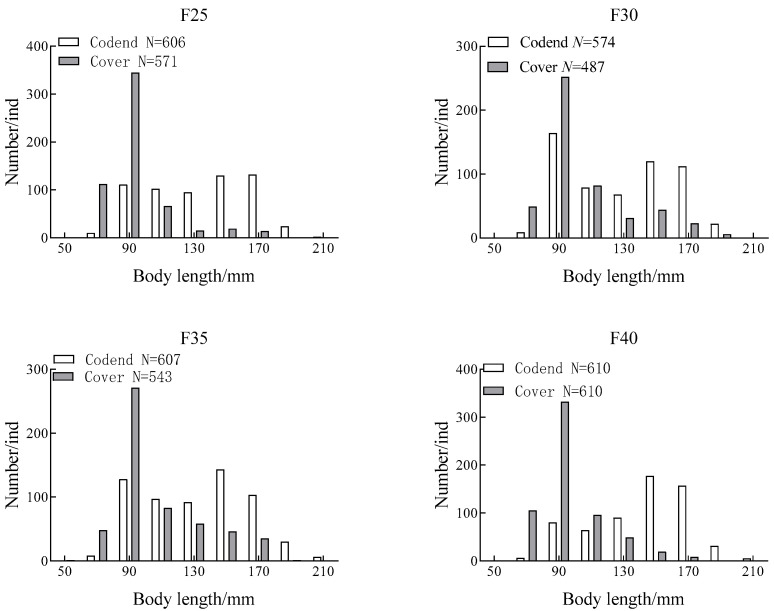
Distribution of body length of *C*. *mystus* in the square mesh codend and cover net.

During the experiment, a total of 4608 individuals of *C*. *mystus* were measured. The length-frequency distributions of *C. mystus* caught by the experimental codends and cover nets with mesh sizes of 25 mm, 30 mm, 35 mm, and 40 mm are shown in Figure 3.

In the 25 mm codend, the mean total length of *C. mystus* was 132.42 mm, with dominant length groups of 140–180 mm. In the corresponding cover net, the mean total length was 91.65 mm, and the dominant length group was 80–100 mm. For the 30 mm codend, the mean length was 127.30 mm, with dominant groups at 80–100 mm and 140–180 mm, while the cover net had a mean of 101.77 mm and a dominant group of 80–100 mm. For the 35 mm codend, the mean length was 131.31 mm, with dominant groups at 80–100 mm and 140–180 mm, while the cover net had a mean of 103.98 mm and a dominant group of 80–100 mm. For the 40 mm codend, the mean length was 140.06 mm, with a dominant group of 140–180 mm, while the cover net had a mean of 94.60 mm and a dominant group of 80–100 mm. The one-way ANOVA showed no significant difference in mean length between the codend and cover samples (*p* > 0.05).

Analysis of the length-frequency distributions indicated that *C. mystus* in the codends were more broadly distributed in size, ranging from 80 to 180 mm, whereas individuals in the cover nets were concentrated mainly in the 80–100 mm range. Comparison among codends with different mesh sizes showed that the mean length of *C. mystus* retained in the codend increased with mesh size, although the dominant length groups remained largely unchanged. A similar trend was observed for fish in the cover nets, where mean length slightly increased with mesh size, but the dominant length group (80–100 mm) remained consistent.

### 3.3. Estimation of Selectivity Parameters

**Figure 4:**
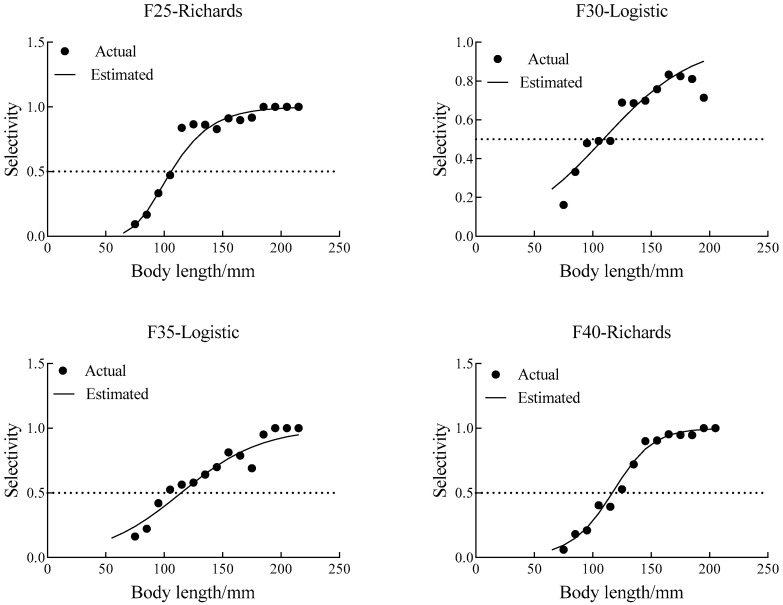
Selectivity curves of the square mesh codend for *C. mystus.* (The dotted line is the 50% selection rate line, with its intersection with the selectivity curve representing the L50).

The selectivity parameters and indices estimated from the models are presented in Table 3. And the selectivity curves are shown in Figure 4. For the F25 codend, the Richards model exhibited smaller residuals and a lower AIC value than the Logistic model, indicating a better overall fit. For the F30 and F35 codends, the Logistic model produced smaller residuals than the Richards model, although the AIC values were slightly higher. In the F30 codend, the difference between the two models was not statistically significant (*p* = 0.101 > 0.05); therefore, the Logistic model was selected as the best-fitting model. For the F35 codend, however, the difference was significant (*p* < 0.05). Considering that the Logistic model had substantially smaller residuals while both models exhibited similar AIC values, the Logistic model was adopted for F35 as well. For the F40 codend, the Richards model showed smaller residuals but a higher AIC value than the Logistic model. Since the difference between models was not significant (*p* = 0.256 > 0.05), the Richards model was selected as the best-fitting model for this mesh size.

**Figure 5:**
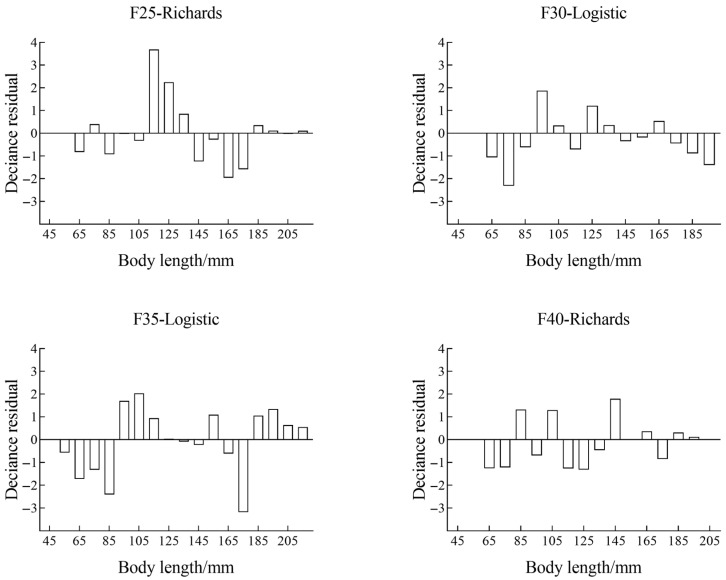
Model deviance residual of the square mesh codend for *C. mystus*.

Comparing the different square-mesh codends, the model fits for F30 and F40 were generally satisfactory (*p* > 0.05), whereas the fits for F25 and F35 showed significant deviations (*p* < 0.05). However, examination of the residuals by fish length (Figure 5) revealed no systematic patterns, suggesting that the poor fit was mainly due to overdispersion rather than structural model defects. Therefore, the estimated selectivity parameters remain valid for reference.

The estimated L50 values for the 25, 30, 35, and 40 mm mesh codends were 105.83 mm, 109.02 mm, 114.36 mm, and 116.66 mm, respectively. The corresponding selection ranges (SR) were 36.95 mm, 84.99 mm, 75.26 mm, and 38.82 mm. As mesh size increased, the L50 of *C. mystus* also increased, and the selectivity curve shifted toward larger body lengths, indicating improved selection performance. However, SR did not increase consistently with mesh size. The F30 and F35 codends exhibited broader SR values, suggesting flatter selectivity curves, whereas the F25 and F40 codends had narrower SR values, indicating steeper curves and stronger selectivity. 

### 3.4. Relationship Between Codend Mesh Size and L50

To determine an appropriate minimum retention length, a linear regression analysis was performed between mesh size and the L50 based on the estimated selectivity parameters. The relationship between L50 and mesh size for *C. mystus* was expressed as L50 = 0.757 *m* + 86.880 (*R*^2^ = 0.9790). According to this regression, L50 increased linearly with increasing mesh size, indicating strong geometric similarity. Based on the reference minimum landing length of *C. mystus* (120 mm) [27], the corresponding minimum mesh size for the square-mesh codend suitable for *C. mystus* in stownet fisheries was estimated to be 43.81 mm.

### 3.5. Escapement Rate and Proportion of Juveniles

During the experiment, the total catch escapement rates calculated by weight for all species were 27.92%, 46.74%, 52.31%, and 55.49% for the 25 mm, 30 mm, 35 mm, and 40 mm square-mesh codends, respectively. The overall escapement rate increased with increasing mesh size.

In this study, juvenile *C. mystus* were defined as individuals with a body length below the minimum landing size of 120 mm. Table 4 summarizes the proportions of juveniles in both the codend and the cover net. The proportions of juvenile *C. mystus* retained in the codend and collected in the cover net were statistically compared using one-way ANOVA, which indicated a significant difference between the two samples (*p* < 0.05). Referring to the optimal minimum landing length proposed by Chen [27], the proportion of juveniles in the codends generally decreased with increasing mesh size, while the proportion of juveniles in the cover nets did not show a consistent increasing trend. The lowest proportion of juveniles in the codend was observed in the F40 codend (24.59%), whereas the highest proportion in the cover net occurred in the F25 codend. This discrepancy may be attributed to variability among fishing operations. Notably, the juvenile proportion in the cover net corresponding to the F40 codend was still as high as 87.38%, confirming the effective size-selective performance of the larger mesh. The selectivity curve of F40 (Figure 4) further indicates that selectivity had been substantially optimized, allowing more efficient escapement of juveniles while maintaining the retention of target-sized fish.

## 4. Discussion

Currently, coastal stownet fisheries generally suffer from poor selectivity and small mesh sizes, which seriously constrain their sustainable development [28,29,30]. To address this issue, a mesh selectivity experiment with diamond-shaped codends of different mesh sizes was conducted in the South China Sea in 2015, yielding encouraging results [4]. Building on that work, the present study conducted the first selectivity trials on *C*. *mystus* using 4 square-mesh codends. The results showed that the L50 increased with mesh size, while the proportion of juveniles declined markedly, indicating that larger mesh sizes improve gear selectivity. However, when the mesh size reached 40 mm, the L50 was 116.66 mm, which was still below the legal minimum landing length (120 mm), suggesting that further optimization of square-mesh codends is required.

Huang et al. [13] reported that, for small yellow croaker (*Larimichthys polyactis*) in stownet, satisfactory selectivity could be achieved with a 50 mm diamond mesh or a 40 mm square mesh. Nevertheless, small yellow croaker is an elongated species, whereas *C. mystus* is laterally compressed. Previous studies have indicated that square meshes generally perform better for cylindrical or elongated species, whereas diamond meshes are more suitable for laterally compressed fish. Therefore, although the 40 mm square mesh provides good selectivity for small yellow croaker, its performance for *C. mystus* remains suboptimal.

The experiment was conducted in September, and the size composition of *C. mystus* was similar to that recorded in August 2015 but smaller than that observed during the spring trial, suggesting seasonal differences in population size structure rather than changes in resource abundance [5]. The linear regression between L50 and mesh size yielded a minimum mesh size of 43.81 mm, which is consistent with the results of diamond-mesh studies in 2015 (42.95 mm in spring and 43.87 mm in summer), suggesting that the potential for further improving *C. mystus* selectivity with square meshes is limited.

Nevertheless, this study systematically revealed the effects of mesh size on the selectivity of *C. mystus*, providing a scientific basis for optimizing codend design and improving the resource efficiency of stownet operations. Proper adjustment of mesh size can effectively enhance gear selectivity and promote the sustainable development of coastal stownet fisheries. The wider selection ranges observed in the 30 mm and 35 mm codends (SR = 84.99 mm and 75.26 mm) may be explained by localized mesh deformation occurring when mesh size approaches the threshold at which fish can nearly pass through. Under such conditions, fish are more likely to struggle and apply uneven pressure to the mesh, increasing deformation variability and thereby widening the observed selection range [31,32].

As *C. mystus* is a major commercial species and a key target of stownet fisheries in the South China Sea, establishing appropriate minimum mesh size standards is of great management and ecological importance. While larger mesh sizes effectively increase juvenile escapement and contribute to resource conservation, they may also reduce the retention of marketable-sized *C. mystus*, potentially affecting economic returns for fishers. Therefore, selecting an appropriate mesh size requires balancing ecological benefits with fishery productivity. In this context, the estimated optimal mesh size (~44 mm) may provide a practical compromise, as it improves selectivity for juvenile fish while still retaining a reasonable proportion of commercially valuable individuals. Such trade-off considerations are essential for developing management measures that promote long-term sustainability without imposing disproportionate economic losses on the fishing industry. This study fills an important gap in knowledge regarding the selectivity of *C. mystus* in square meshes and provides baseline data for resource conservation and gear management. Future research should focus on refining the matching between mesh shape and size, exploring the potential of composite mesh structures for laterally compressed species, and integrating behavioral observations to better understand the escapement mechanisms of *C. mystus*.

To address limitations of the 2015 experiments, this study employed iron rings to reduce the covering effect of the cover net on the inner codend. Although this improvement enhanced codend expansion, differences in ring size may still have affected the effective mesh opening area, leading to potential bias in parameter estimation. Further studies should expand the range of square mesh sizes, explore the combined effects of mesh shape and codend structure, and integrate fish behavior analysis to elucidate escapement processes more comprehensively.

In addition, Mituhasi et al. [33] obtained that the maximum body girth is a key geometric factor determining whether fish can pass through a mesh opening. When the girth-to-mesh perimeter ratio was slightly larger than 1, the retention rate could reach nearly 100%. However, no published data are currently available on the maximum body girth or body depth of *C. mystus* in the South China Sea [34]. Therefore, although this study demonstrated that the improvement in selectivity provided by square mesh codends remains limited, it was not possible to quantitatively evaluate the girth-to-mesh perimeter relationship and its linkage to size selectivity. This aspect will be further investigated in future research.

## 5. Conclusions

In summary, our study showed that the size selectivity of *C.mystus* in stownet improved significantly with increasing square mesh size. The optimal minimum mesh size was estimated at 43.81 mm. However, the L50 value for the largest tested mesh (40 mm) remained below the legal minimum landing length (120 mm), indicating that the improvement in selectivity of square-mesh codends for *C. mystus* is limited. These findings provide a scientific basis for optimizing net design and refining mesh size regulations in South China Sea stownet fisheries.

## Figures and Tables

**Table 1:** Specifications of the experimental square-mesh codends and cover net used in the selectivity experiments.

Codend ID	Mesh Size (mm)	Effective Operation	Mesh Shape	No. of Meshes (L)	Length (m)	No. of Meshes (W)	Width (m)	Netting Structure
F25	25	6	Square section	196	2.45	84	3.0	2 × 3
			Diamond section	10	0.25	120.5	3.0	2 × 3
F30	30	6	Square section	160	2.40	70	3.0	2 × 3
			Diamond section	10	0.30	100	3.0	2 × 3
F35	35	6	Square section	134	2.35	60	3.0	2 × 3
			Diamond section	10	0.35	85.5	3.0	2 × 3
F40	40	6	Square section	115	2.30	52.5	3.0	2 × 3
			Diamond section	10	0.40	75	3.0	2 × 3
Cover net	15	-	Diamond	270	4.05	240	3.6	2 × 3

Notes: L = longitudinal direction; W = transverse direction; All codends were made of polyethylene (PE) twine with a structure of 36 tex × 2 × 3.

**Table 2:** Catch Composition of Different Mesh Sizes in Codend and Covered Net.

Mesh Size/mm	Codend/Covered Net	Species	*F*/%	*N*/%	*W*/%	IRI
25	Codend	*C. mystus*	100.00	85.96	72.62	15,857.92
		*Collichthys lucidus*	100.00	10.92	14.89	2581.08
		Other species		3.12	12.49	
	Cover net	*C. mystus*	100.00	87.31	78.84	16,614.39
		*Odontamblyopus rubicundus*	83.33	7.19	10.63	1484.67
		Other species		5.50	10.54	
30	Codend	*C. mystus*	100.00	76.33	65.10	14,143.37
		*C. lucidus*	100.00	17.42	22.78	4020.45
		Other species		6.25	12.11	
	Cover net	*C. mystus*	100.00	79.27	74.75	15,402.27
		*O. rubicundus*	66.67	10.59	11.57	1477.09
		Other species		10.14	13.68	
35	Codend	*C. mystus*	100.00	77.32	55.91	13,323.30
		*C. lucidus*	100.00	8.79	11.62	2040.62
		*Arius sinensis*	100.00	4.97	11.29	1626.15
		Other species		8.92	21.18	
	Cover net	*C. mystus*	100.00	77.24	72.86	15,010.40
		*O. rubicundus*	100.00	11.52	14.69	2620.88
		Other species		6.97	9.50	
40	Codend	*C. mystus*	100.00	85.67	64.01	14,968.87
		*C. lucidus*	100.00	5.06	5.63	1068.75
		Other species		9.27	30.35	
	Cover net	*C. mystus*	100.00	81.12	72.42	15,354.16
		*O. rubicundus*	100.00	14.63	21.33	3595.46
		Other species		4.26	6.25	

**Table 3:** Selective parameters and indexes of the square mesh codend for *C. mystus*.

Parameters	F25		F30		F35		F40	
	Logistic	Richards *	Logistic *	Richards	Logistic *	Richards	Logistic	Richards *
a	−6.055 ± 0.349	−1.434 ± 5.262	−2.819 ± 0.258	−0.001 ± 9.460	−3.339 ± 0.264	−0.010 ± 8.418	−6.577 ± 0.343	−7.352 ± 1.781
b	0.558 ± 0.033	0.435 ± 0.051	0.259 ± 0.022	0.211 ± 0.073	0.292 ± 0.022	0.233 ± 0.060	0.567 ± 0.030	0.606 ± 0.094
δ		0.060 ± 0.277		0.143 ± 1.175		0.103 ± 0.769		1.218 ± 0.501
L50	10.848 ± 0.137	10.583 ± 0.158	10.902 ± 0.259	10.742 ± 0.364	11.436 ± 0.223	11.231 ± 0.302	11.594 ± 0.138	11.666 ± 0.211
SR	3.936 ± 0.232	3.695 ± 0.485	8.499 ± 0.728	7.843 ± 12.513	7.526 ± 0.570	7.011 ± 6.331	3.873 ± 0.203	3.882 ± 0.471
*H*_0_: Model fit								
DR	47.283	29.303	15.950	18.636	34.17	99.02	16.01	14.72
DOF	14	13	12	11	15	14	13	12
*p*-value	0.000	0.006	0.194	0.068	0.003	0.000	0.249	0.257
*H*_0_: *δ* = 1								
DR		17.98		2.69		64.85		1.29
DOF		1		1		1		1
*p*-value		0.000		0.101		0.000		0.256
AIC	98.06	82.90	76.06	75.17	92.38	88.71	69.18	71.00

Note: a, b, and *δ* is the model parameters; L50 is the fish length at 50% retention probability, SR is the selection range; DR represents value of model deviance; DOF represents the degree of freedom; AIC is the Akaike Information Criterion; * indicates that the curve will be applied.

**Table 4:** The proportions of juveniles *C. mystus* in both the codend and the cover net.

Codend/ Cover Net	Optimum Fist Capture Length/mm	Proportions of Juveniles *C. mystus* in F25/%	Proportions of Juveniles *C. mystus* in F30/%	Proportions of Juveniles *C. mystus* in F35/%	Proportions of Juveniles *C. mystus* in F40/%
Codend	120	36.80	43.90	38.39	24.59
Cover net	120	91.59	78.64	74.22	87.38

## Data Availability

The original contributions presented in this study are included in the article. Further inquiries can be directed to the corresponding author.
